# Biosynthesis of Selenocysteine on Its tRNA in Eukaryotes

**DOI:** 10.1371/journal.pbio.0050004

**Published:** 2006-12-26

**Authors:** Xue-Ming Xu, Bradley A Carlson, Heiko Mix, Yan Zhang, Kazima Saira, Richard S Glass, Marla J Berry, Vadim N Gladyshev, Dolph L Hatfield

**Affiliations:** 1 Molecular Biology of Selenium Section, Laboratory of Cancer Prevention, Center for Cancer Research, National Cancer Institute, National Institutes of Health, Bethesda, Maryland, United States of America; 2 Department of Biochemistry, University of Nebraska, Lincoln, Nebraska, United States of America; 3 Department of Chemistry, The University of Arizona, Tucson, Arizona, United States of America; 4 Department of Cell and Molecular Biology, University of Hawaii at Manoa, Honolulu, Hawaii, United States of America; University of Michigan, United States of America

## Abstract

Selenocysteine (Sec) is cotranslationally inserted into protein in response to UGA codons and is the 21st amino acid in the genetic code. However, the means by which Sec is synthesized in eukaryotes is not known. Herein, comparative genomics and experimental analyses revealed that the mammalian Sec synthase (SecS) is the previously identified pyridoxal phosphate-containing protein known as the soluble liver antigen. SecS required selenophosphate and *O*-phosphoseryl-tRNA^[Ser]Sec^ as substrates to generate selenocysteyl-tRNA^[Ser]Sec^. Moreover, it was found that Sec was synthesized on the tRNA scaffold from selenide, ATP, and serine using tRNA^[Ser]Sec^, seryl-tRNA synthetase, *O-*phosphoseryl-tRNA^[Ser]Sec^ kinase, selenophosphate synthetase, and SecS. By identifying the pathway of Sec biosynthesis in mammals, this study not only functionally characterized SecS but also assigned the function of the *O-*phosphoseryl-tRNA^[Ser]Sec^ kinase. In addition, we found that selenophosphate synthetase 2 could synthesize monoselenophosphate in vitro but selenophosphate synthetase 1 could not. Conservation of the overall pathway of Sec biosynthesis suggests that this pathway is also active in other eukaryotes and archaea that synthesize selenoproteins.

## Abstract 2

Sec synthase, a conserved protein responsible for the biosynthesis of the rare 21st amino acid, selenocysteine, is identified in eukaryotes, and the underlying biochemical pathway is characterized.

## Author Summary

Biosynthesis of the 20 canonical amino acids is well established in eukaryotes. However, many eukaryotes also have a rare selenium-containing amino acid, selenocysteine, which is the 21st amino acid in the genetic code. Selenium is essential for human health, and its health benefits, including preventing cancer and heart disease and delaying aging, have been attributed to the presence of selenocysteine in protein. How selenocysteine is made in eukaryotes has not been established. To gain insight into its biosynthesis, we used computational analyses to search completely sequenced genomes for proteins that occur exclusively in organisms that utilize selenocysteine. This approach revealed a putative selenocysteine synthase, which had been previously identified as a pyridoxal phosphate–containing protein dubbed soluble liver antigen. We were able to characterize the activity of this synthase using selenophosphate and a tRNA aminoacylated with phosphoserine as substrates to generate selenocysteine. Moreover, identification of selenocysteine synthase allowed us to delineate the entire pathway of selenocysteine biosynthesis in mammals. Interestingly, selenocysteine synthase is present only in those archaea and eukaryotes that make selenoproteins, indicating that the newly defined pathway of selenocysteine biosynthesis is active in these domains of life.

## Introduction

Selenocysteine (Sec) is a selenium-containing amino acid that is cotranslationally inserted into protein and is recognized as the 21st amino acid in the genetic code [1–3]. Sec is incorporated into protein in all three lines of descent, eukaryota, archaea, and eubacteria, but unlike other amino acids, Sec synthesis occurs on its transfer RNA (tRNA), designated tRNA^[Ser]Sec^ [4,5]. tRNA^[Ser]Sec^ is initially aminoacylated with serine by seryl-tRNA synthetase and the seryl moiety provides the backbone for Sec synthesis. The biosynthesis of Sec was established in Escherichia coli in the early 1990s [6–8]. Bacterial Sec synthase (SecS) (E. coli selenocysteine synthase [SelA]) is a pyridoxal phosphate (PLP)-dependent protein that converts the serine attached to tRNA^[Ser]Sec^ to Sec by initially removing the hydroxyl group from serine to form an aminoacrylyl intermediate. This intermediate serves as the acceptor for activated selenium, and when selenium is donated, selenocysteyl-tRNA^[Ser]Sec^ is formed. The active selenium donor in bacteria is synthesized from selenide and ATP by E. coli selenophosphate synthetase (SelD), and the product of the reaction has been identified as monoselenophosphate (SeP) [9].

A distant homolog of bacterial SelA (SelA-like) is present in some archaea but is not active as SecS [10], and it does not always co-occur in archaea with Sec insertion systems. In addition, no SelA sequences could be detected in eukaryotes. Although Sec insertion systems are different in bacteria from those in archaea and eukaryotes [11–13], several factors have been characterized in mammals that most certainly have a role in Sec biosynthesis. For example, the soluble liver antigen (SLA) was initially identified as a 48-kDa protein bound to Sec tRNA^[Ser]Sec^ that was targeted by antibodies in patients with an autoimmune chronic hepatitis [14]. SLA was subsequently reported to exist as a separate family within a larger superfamily of diverse PLP-dependent transferases [15], and this protein has been proposed to function as the mammalian SecS (e.g., see [3,15–17]). Further evidence that SLA is involved in selenium metabolism is that it was found to occur in a protein complex with other factors involved in the biosynthesis of Sec and/or its insertion into protein [17,18]. In addition, a kinase that phosphorylated a minor seryl-tRNA was reported in 1970 [19] that was subsequently isolated, characterized, and found to specifically phosphorylate the seryl moiety on seryl-tRNA^[Ser]Sec^ [20]. The resulting phosphoseryl-tRNA^[Ser]Sec^ was proposed either as a candidate substrate for SecS (see [3,20] and references therein) or it served as a storage form [21] . Furthermore, two genes initially thought to have a role in selenophosphate synthesis, *sps1* and *sps2*, have been reported in mammals [22–25], and the product of *sps2* is a selenoprotein, SPS2 [22,24]. The Sec-to-Cys mutant form of SPS2 has low enzyme activity [22,24,26] and can complement SelD in Escherichia coli cells transfected with the mammalian mutant form [26]. Complementation of SelD^−^ E. coli cells with SPS1 or SPS2 has suggested that SPS1 may have a role in recycling Sec via a selenium salvage system and SPS2 may be involved in the de novo synthesis of selenophosphate from selenide [27]. However, it should be noted that, to our knowledge, selenophosphate has never been shown to serve directly as the active selenium donor in Sec biosynthesis in eukaryotes.

Herein, we used a comparative genomics search and experimental analyses to show that SLA is the mammalian SecS. This protein belongs to a different family of PLP-containing enzymes and uses *O*-phosphoseryl-tRNA^[Ser]Sec^ rather than seryl-tRNA^[Ser]Sec^ as substrate. SecS dephosphorylates *O*-phosphoseryl-tRNA^[Ser]Sec^ and accepts the active selenium donor to yield selenocysteyl-tRNA^[Ser]Sec^. We also demonstrated unequivocally that the selenium donor in eukaryotes is SeP by using this compound as a substrate in a reaction with SecS and phosphoseryl-tRNA^[Ser]Sec^. Selenophosphate is indeed synthesized in mammals by SPS2, whereas the distant homolog of SelD in mammals, SPS1, did not synthesize the active selenium donor. Conservation of the overall pathway of Sec biosynthesis suggests that it is also active in other eukaryotes and archaea.

## Results

### Computational Search and Comparative Genomics Analysis

**Figure 1:**
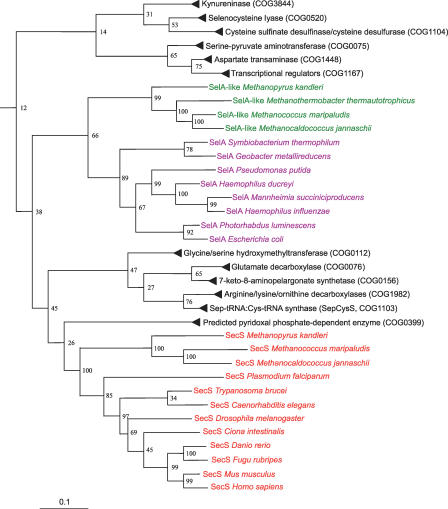
Phylogenetic Tree of SecS, SelA, SelA-Like, and Other PLP-Dependent Proteins SecS proteins are shown in red; SelA in pink, and archaeal SelA-like proteins in green. Other PLP-dependent protein branches are compressed and represented by family names. The phylogenetic tree was generated by ClustalW and PHYLIP programs. Both bootstrap support (the number of times each branch was supported in bootstrap replication) and the measurement of distance for the branch lengths (shown by a bar) are indicated.

**Figure 2:**
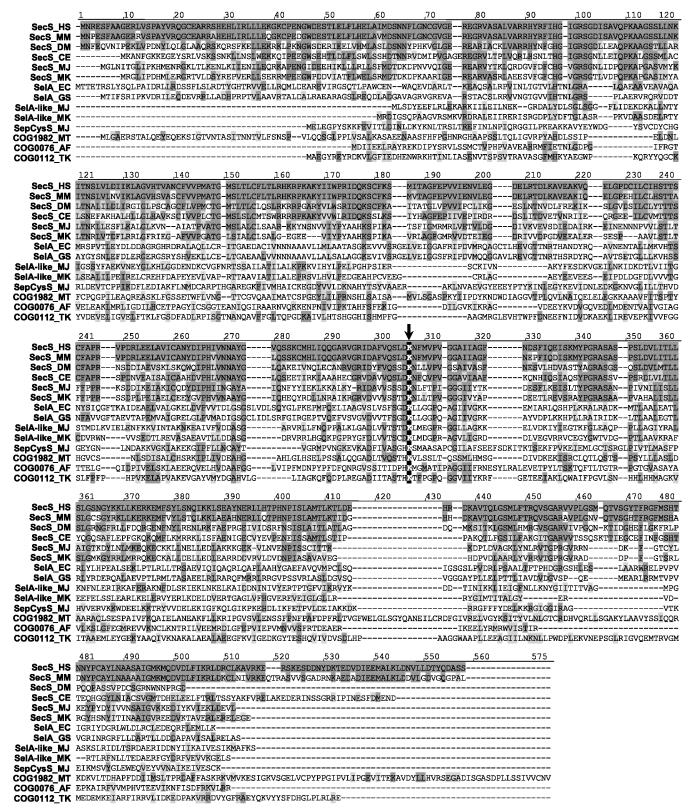
Multiple Alignment of SecS, SelA, SelA-Like, and Other PLP-Dependent Protein Sequences GenBank accession numbers for the sequences are given in the Accession Numbers summary. The active site lysine (K), which is PLP-binding residue, is indicated with an arrow.

We analyzed completely sequenced genomes of eukaryotes and archaea for the occurrence of selenoproteins. Twenty-six eukaryotes and three archaea that had these proteins and 24 eukaryotes and 24 archaea that did not were identified. Comparative genomics studies were then carried out to identify genes that co-occur with selenoproteins in (1) eukaryotes (Table 1) and (2) archaea (Table 2). Each of the searches had known components of Sec insertion machinery as top candidates as well as an additional protein, herein designated as SecS. In mammals, SecS is also known as SLA. SLA was first detected as an autoimmune factor that coimmunoprecipitated tRNA^[Ser]Sec^ from cell extracts in patients with autoimmune chronic hepatitis [14], and it also bound other Sec insertion components [17,18]. SecS formed a separate family within a larger superfamily of diverse PLP-dependent proteins and was previously suggested to convert a tRNA-bound serine to Sec [15]. We found that it occurred exclusively in both eukaryotes and archaea which had selenoproteins but was lacking in the other organisms examined (Figure 1 and Tables 1 and 2). These observations strongly suggested that SecS may be the missing SecS in eukaryotes and archaea. Based on the multiple sequence alignment and phylogenetic analysis of SecS and other PLP-dependent proteins, including SelA, SelA-like, and SepCysS, it was clear that bacterial SelA and archaeal SelA-like proteins [10], on one hand, and SecS, on the other, belonged to completely different families of PLP-containing proteins (Figures 1 and 2), suggesting that their similar functions arose by convergent evolution. SecS was also distantly homologous to SepCysS, a protein recently found to synthesize cysteine from phosphoserine in some archaea [28] (Figures 1 and 2). After identifying a likely SecS candidate by comparative genomics analysis, we experimentally verified its function as described below.

**Table 1:**
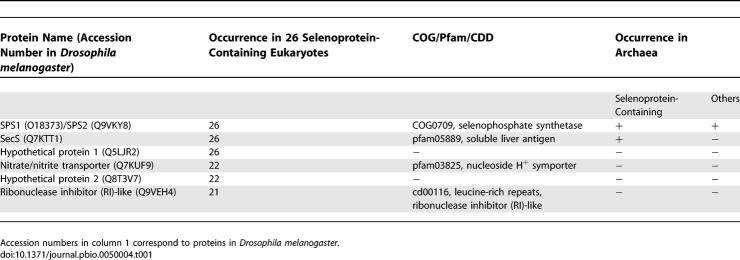
Phylogenetic Profiles of Eukaryotic Proteins That Are Present Exclusively in Selenoprotein-Containing Eukaryotes

**Table 2:**
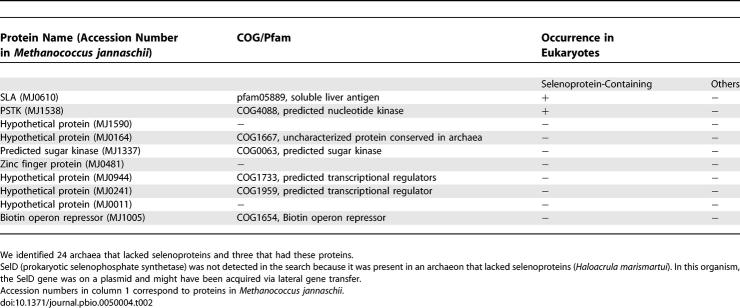
Phylogenetic Profiles of Archaeal Proteins That Are Present Exclusively in Selenoprotein-Containing Archaea

### Binding and Dephosphorylation of *O*-phosphoseryl-tRNA^[Ser]Sec^ by Mouse SecS

**Figure 3:**
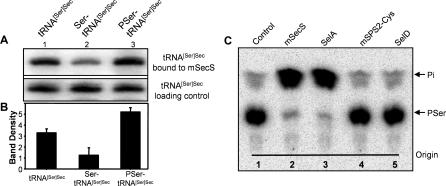
Binding of tRNA^[Ser]Sec^ to mSecS-Cys and Dephosphorylation of *O*-Phosphoseryl-tRNA^[Ser]Sec^ by mSecS-Cys (A) Relative efficiencies of tRNA^[Ser]Sec^, seryl-tRNA^[Ser]Sec^, and *O-*phosphoseryl-tRNA^[Ser]Sec^ binding to mSecS are shown. Cloning of mSecS-Cys, its expression and purification, binding assays, and Northern analysis are detailed in Materials and Methods. The upper panel shows the binding of each form of tRNA^[Ser]Sec^ to mSecS-Cys. The lower panel shows the amount of each tRNA^[Ser]Sec^ used in the binding reaction which was assessed by Northern blot analysis of 2 μl of each binding reaction solution designated as tRNA loading control. (B) Amounts of each tRNA^[Ser]Sec^ form bound to mSecS in (A) above were quantified by measuring the band densities of each form bound to mSecS relative to those of the corresponding densities of the amount of each form added to the assay using ImageQuant Version 5.2 (Molecular Dynamics). The error bars were derived from four separate binding assays. The *p*-value in each case is <0.01. (C) ^32^P-labeled *O*-phosphoseryl-tRNA^[Ser]Sec^ was added to a reaction mixture containing either mSecS, SelA, mSPS2-Cys, or SelD, and the reaction was incubated, the aminoacyl-tRNA^[Ser]Sec^ was deacylated, and the deacylated products were chromatographed as given in Materials and Methods. Lane 1 contains ^32^P-labeled *O*-phosphoserine, and the other lanes contain the components shown.

To elucidate Sec biosynthesis in mammals, we initially examined the ability of tRNA^[Ser]Sec^, seryl-tRNA^[Ser]Sec^, and *O-*phosphoseryl-tRNA^[Ser]Sec^ to bind to the recombinant mouse SecS (mSecS). The coprecipitated product was detected by Northern blotting (Figure 3A) and the amount of binding was quantitated (Figure 3B). *O*-Phosphoseryl-tRNA^[Ser]Sec^ bound more efficiently to mSecS than the other tRNA^[Ser]Sec^ forms, while seryl-tRNA^[Ser]Sec^ bound least efficiently, suggesting that *O-*phosphoseryl-tRNA^[Ser]Sec^ may be a substrate for mSecS. It is not clear why tRNA^[Ser]Sec^ binds to mSecS (see also [18]), albeit less efficiently than *O*-phosphoseryl-tRNA^[Ser]Sec^. Seryl-tRNA^Ser^ and tRNA^Ser^, however, did not manifest any binding to mSecS (unpublished data).

To assess whether the phosphate moiety on *O*-phosphoseryl-tRNA may be removed by mSecS to generate an intermediate that serves as an acceptor for the active selenium donor, the ^32^P-labeled form of *O-*phosphoseryl-tRNA^[Ser]Sec^ was incubated with mSecS (Figure 3C). mSecS removed the phosphoryl moiety from *O-*phosphoseryl-tRNA^[Ser]^ ^Sec^ (see lane 2). Interestingly, SelA was also capable of dephosphorylating *O*-phosphoseryl-tRNA^[Ser]Sec^ (lane 3). Neither mSPS2-Cys [mouse selenophosphate synthetase 2 containing an Sec (UGA)-to-Cys (UGC) mutation] nor SelD appeared to have any effect on *O-*phosphoseryl-tRNA^[Ser]Sec^ (lanes 4 and 5). The dephosphorylation of *O*-phosphoseryl-tRNA^[Ser]Sec^ by mSecS and SelA is further considered below. However, it should be noted that the data in Figure 3 strongly suggest that the dephosphorylated product is not seryl-tRNA^[Ser]Sec^ as the product binds efficiently to mSecS but seryl-tRNA does not (Figure 3B).

### In Vitro ATP Hydrolysis Assay of Selenophosphate Synthetase and NMR Spectroscopic Analysis

**Figure 4:**
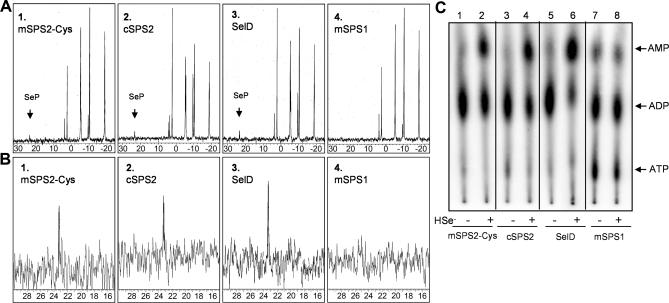
In Vitro ATP Hydrolysis Assay of Selenophosphate Synthetase and NMR Spectroscopic Analysis Cloning of the genes, mouse *sps1,* mouse *sps2, SelD,* and *C. elegans sps2,* and mutation of mouse *sps2* to *sps2-Cys* and reaction conditions are detailed in Materials and Methods. For NMR analysis, 200 μl of ATP hydrolysis reaction was sealed in 3-mm NMR tubes and incubated at 37 °C for 4 h before ^31^P NMR spectroscopic analysis [9]. (A) Complete ^31^P-NMR spectra of ATP hydrolysis products generated with mSPS2-Cys, C. elegans selenophosphate synthetase 2, SelD, and mSPS1 are shown. (B) Expanded spectra of the ordinate and abscissa between 15 and 30 ppm for these products are shown. (C) ATP hydrolysis reactions with [α-^32^P]ATP, either without or with 0.25 mM selenide, incubated with mSPS2-Cys, C. elegans selenophosphate synthetase 2, SelD, or mSPS1; at the end of the incubation period, reactions were loaded onto PEI TLC plates, run in 0.8 M LiCl, and exposed to a PhosphorImager screen as described in Materials and Methods.

We next identified the active selenium donor by assessing whether mSPS1 and mSPS2-Cys synthesized SeP. ^31^P NMR spectroscopic analysis of the products of the mSPS2-Cys–catalyzed reaction manifested a signal at +23.2 ppm, albeit weakly (Figure 4A1), that corresponded to SeP [9,29]. Since the mSPS2 used in this experiment was a Sec-to-Cys mutant and might not be expected to generate SeP efficiently, we cloned SPS2 from *Caenorhabditis elegans,* which naturally contains Cys in place of Sec at the presumed active site of SPS2 [13]. C. elegans SPS2 clearly generated a signal at +23.2 ppm (Figure 4A2). As expected, SeP was also formed in the presence of E. coli SelD, selenide, and ATP (Figure 4A3) [9,29]. However, no signal at +23.2 ppm was observed when mSPS1 replaced mSPS2-Cys or SelD in the reaction, indicating that mSPS1 did not synthesize SeP (Figure 4A4).

As the peak at +23.2 ppm was relatively weak in the product analysis of mSPS2-Cys (Figure 4A1), the ordinate and abscissa of the area between 15 and 30 ppm were expanded as shown in Figure 4B. Clearly, there was a peak at +23.2 ppm corresponding to SeP, demonstrating that mSPS2-Cys produced SeP. The signal for SeP was also evident with C. elegans SPS2 and with SelD, but mSPS1 did not produce this signal.

To further examine the hydrolysis of ATP by mSPS2-Cys, C. elegans SPS2, SelD, and mSPS1, each component was incubated with [α-^32^P]ATP with and without selenide (Figure 4C). Hydrolysis of ATP to AMP was largely dependent on the presence of selenide with the three enzymes, mSPS2-Cys, C. elegans SPS2, and SelD, that produced SeP (see above), and all three hydrolyzed ATP to ADP independently of selenide. Although mSPS1 hydrolyzed ATP to ADP and apparently only slightly to AMP, this degradation was independent of selenium. These data provide further evidence that mSPS1 cannot synthesize SeP from selenide.

### In Vitro Sec Biosynthesis

**Figure 5:**
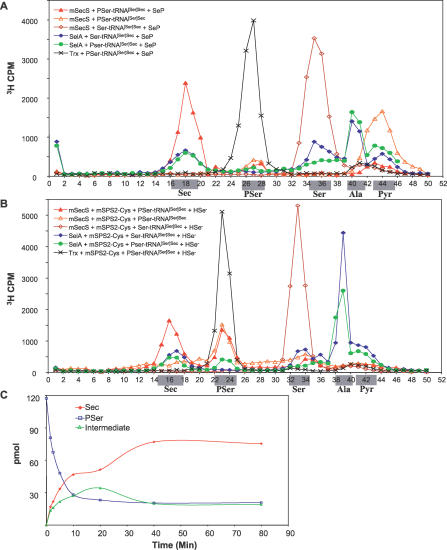
In Vitro Sec Biosynthesis All reactions were carried out under anaerobic conditions and are detailed in Materials and Methods. Synthetic tRNA^[Ser]Sec^ was used in those reactions employing tRNA and its synthesis, aminoacylation with ^3^H-serine and phosphorylation were carried out as given [20]. Cloning of mouse *sps2*, and preparation of the Sec-to-Cys *sps2* mutant, and cloning of *E. coli SelA* and mouse *SecS* are given in Materials and Methods. (A) Sec biosynthesis using SeP as the active selenium donor with *O*-phosphoseryl-tRNA^[Ser]Sec^ or seryl-tRNA^[Ser]Sec^ and either mSecS or SelA as SecS is shown. (B) Sec biosynthesis using mSPS2-Cys and selenide (HSe^−^) to provide SeP as the active selenium donor with *O*-phosphoseryl-tRNA^[Ser]Sec^ or seryl-tRNA^[Ser]Sec^ and either mSecS or SelA as SecS is shown. HSe^−^ was maintained in the reduced state in reactions in B with DTT as described in Materials and Methods. Migration of control amino acids and pyruvate are indicated below the graphs in (A) and (B). (C) The rate of Sec synthesis is shown. Reactions were terminated at 0, 1.25, 2.5, 5, 10, 20, 40, and 80 min. After chromatography and counting of samples in a liquid scintillation counter as given in Materials and Methods, the counts from the peaks of Sec, *O*-phosphoserine, or the degraded intermediate (the peak migrated after alanine and chromatographed with pyruvate) were pooled together for quantification at each time point. The analyses in this figure were carried out on deacylated products.

Previous studies analyzing Sec biosynthesis did not utilize SeP to assess whether this compound served directly as the active selenium donor. We therefore examined the ability of SeP to donate selenium directly in Sec biosynthesis. Sec was indeed synthesized when SeP [9,29] was added in the reaction with *O-*phosphoseryl-tRNA^[Ser]Sec^ and mSecS (Figure 5A). This observation confirms unequivocally that SeP is the active donor of selenium in Sec biosynthesis and that SecS is the missing SecS. Control assays demonstrated that Sec was not formed when SeP was omitted from the reaction, when seryl-tRNA was used in place of *O-*phosphoseryl-tRNA^[Ser]Sec^, or when another protein, thioredoxin (Trx), was substituted for mSecS in the reaction. As expected, a reaction consisting of SelA, seryl-tRNA^[Ser]Sec^, and SeP also synthesized Sec (Figure 5A). *O*-Phosphoseryl-tRNA^[Ser]Sec^ could also serve as a substrate and replace seryl-tRNA^[Ser]Sec^ in reactions with SelA and SeP, thus using the dephosphorylated product as an acceptor for activated selenium to synthesize selenocysteyl-tRNA^[Ser]Sec^.

Sec was also synthesized on tRNA^[Ser]Sec^ when *O-*phosphoseryl-tRNA^[Ser]Sec^ was incubated with mSecS, mSPS2-Cys, ATP, and selenide (Figure 5B). Control reactions demonstrated that Sec was not formed when selenide was omitted from the reaction, when seryl-tRNA^[Ser]Sec^ was used in place of *O-*phosphoseryl-tRNA^[Ser]Sec^, or when Trx was substituted for mSecS (Figure 5B). SelA would substitute for mSecS when the substrate was seryl-tRNA^[Ser]Sec^ or *O*-phosphoseryl-tRNA^[Ser]Sec^.

As expected, SelD could substitute for mSPS2-Cys in synthesizing SeP and generating similar amounts of Sec as mSPS2-Cys in reactions with mSecS (unpublished data), and those reactions in Figure 5B were dependent on ATP as well as selenide wherein Sec was generated (unpublished data).

Serine, alanine, and pyruvate were recovered from reactions with SelA using either seryl- or *O*-phosphoseryl-tRNA^[Ser]Sec^ as substrates, wherein alanine and pyruvate were likely the deacylated, degraded products of the intermediate, aminoacrylyl-tRNA^[Ser]Sec^ [7] (Figures 5A and 5B). In reactions with mSecS using *O*-phosphoseryl-tRNA^[Ser]Sec^ as substrate, only a small amount of phosphoserine and a peak that comigrated with pyruvate were recovered as deacylated products, suggesting that pyruvate was, similar to the bacterial case, the deacylated, degraded product of the intermediate in Sec biosynthesis in eukaryotes. However, the amount of pyruvate recovered in reactions that were coupled with SeP synthesis by selenophosphate synthetase was lower (Figure 5B) than in reactions in which SeP was supplied directly as substrate (Figure 5A). Use of HSe^−^ in these reactions required the addition of high levels of dithiothreitol (DTT) which were inhibitory to Sec synthesis (unpublished data), and apparently the intermediate that formed pyruvate as a deacylated product was unstable under these conditions. The intermediates in reactions with SelA [7] and mSecS are further considered in Discussion.

We also examined the rate of Sec synthesis with *O*-phosphoseryl-tRNA^[Ser]Sec^ and SeP as substrates in the presence of mSecS (Figure 5C). As the substrate, *O*-phosphoseryl-tRNA^[Ser]Sec^, was labeled with [^3^H]serine and the deacylated products, *O*-phosphoserine, Sec and the degraded intermediate, pyruvate, migrated separately in the chromatographic system used in Figure 5A; the amounts of each could be assessed during the course of the reaction. Dephosphorylation occurred rapidly and appeared to be near completion in about 10 min. Sec synthesis increased rapidly during the initial 10 min and then appeared to proceed more slowly until completion at about 40 min. The approximate initial rate was 0.28 pmol Sec/min/pmol mSecS. Likewise, the intermediate formed rapidly during the initial stages of the reaction and then decreased over the remainder of the experiment.

## Discussion

In this work, we defined the pathway of Sec biosynthesis in eukaryotes. In order to carry out Sec biosynthesis, we functionally characterized two previously known enzymes, selenophosphate synthetase [22,23,25] and *O-*phosphoseryl-tRNA^[Ser]Sec^ kinase [20], as well as establishing the function of SLA [14] as the eukaryotic SecS. All these enzymes were found to be required for in vitro biosynthesis of Sec, and the implications of these findings are discussed below.

The active selenium donor in bacteria is SeP [9,29] and it is synthesized from selenide and ATP by selenophosphate synthetase, also known as SelD [7,30]. Two homologs of bacterial SelD, designated SPS1 and SPS2, are present in mammals [22–25]. Interestingly, SPS2 is a selenoprotein. Direct roles of SPS1 and SPS2 in mammals have not been tested, but it was suggested that SPS2 supports the use of selenite, whereas SPS1 depends on a selenium salvage system when examined in E. coli [27]. Our results clearly demonstrate that SPS2 makes the active selenium donor, SeP, for the biosynthesis of Sec.

We recently identified the gene that phosphorylates seryl-tRNA^[Ser]Sec^ and characterized the gene product, *O-*phosphoseryl-tRNA^[Ser]Sec^ [20]. However, the precise role of *O-*phosphoseryl-tRNA^[Ser]Sec^ was not determined. The current results showed that *O-*phosphoseryl-tRNA^[Ser]Sec^ is the substrate of SecS, and therefore *O-*phosphoseryl-tRNA^[Ser]Sec^ kinase is involved in the Sec biosynthesis pathway.

What is the intermediate produced by mSecS? Most certainly the dephosphorylated product of *O*-phosphoseryl-tRNA^[Ser]Sec^ cannot be seryl-tRNA^[Ser]Sec^ (Figure 3A). The intermediate could possibly be aminoacrylyl-tRNA^[Ser]Sec^ (dehydroalanyl-tRNA^[Ser]Sec^) which could yield pyruvate on hydrolysis. The facts that the intermediate generated by SelA is aminoacrylyl-tRNA^[Ser}Sec^ and that mSecS contains pyridoxal phosphate suggest that the Schiff base intermediate of aminoacrylyl-tRNA^[Ser}Sec^ postulated for Sec synthesis in prokaryotes [7] is analogous to that formed in eukaryotes. However, it proved possible to trap the proposed aminoacrylyl-tRNA^[Ser]Sec^ by reduction with KBH4 leading to the formation of alanine on hydrolysis in the prokaryotic, but not eukaryotic, case. This result may be due to differences between the enzyme to which the aminoacrylyl-tRNA^[Ser]Sec^ is bound; that is, reduction can occur before hydrolysis in the prokaryotic, but not eukaryotic, case. Nevertheless, identification of the intermediate in Sec biosynthesis in eukaryotes must await further study.

**Figure 6:**
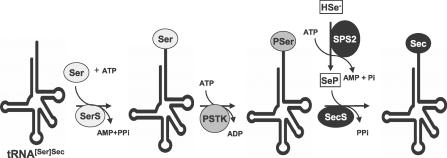
Sec Biosynthesis in Eukaryotes The pathway of Sec biosynthesis is shown (see text for details and abbreviations are defined in the text with the exception of SerS [seryl-tRNA synthetase] and PSTK [*O*-phosphoseryl-tRNA^[Ser]Sec^ kinase]).

The biosynthesis of Sec in eukaryotes is shown in Figure 6. tRNA^[Ser]Sec^ is aminoacylated by seryl-tRNA synthetase and the seryl moiety is phosphorylated by *O-*phosphoseryl-tRNA^[Ser]Sec^ kinase to form *O-*phosphoseryl-tRNA^[Ser]Sec^ [20]. *O-P*hosphoseryl-tRNA^[Ser]Sec^ is a substrate for SecS which replaces the phosphoryl moiety of phosphoserine, derived from the selenium donor, SeP, to yield Sec. SeP is synthesized by SPS2 in the ATP-dependent reaction. SecS does not use seryl-tRNA^[Ser]Sec^ as a substrate (Figure 5A and 5B). Although no enzyme comparable to *O-*phosphoseryl-tRNA^[Ser]Sec^ kinase has been identified in *E. coli,* it is of interest to note that SelA can utilize *O-*phosphoseryl-tRNA^[Ser]Sec^ as a substrate. The major difference between the Sec biosynthetic pathway characterized herein and that in eubacteria is the extra step in the synthesis of *O-*phosphoseryl-tRNA^[Ser]Sec^ which serves as a substrate for SecS. In E. coli, seryl-tRNA^[Ser]Sec^ serves directly as the substrate for SelA [7]. The occurrence of SecS exclusively in selenoprotein-containing organisms in eukaryotes and archaea (Tables 1 and 2) indicates that the SecS-based pathway also operates in other animals, lower eukaryotes, and archaea where the Sec machinery occurs [3]. Considering the difficulties with identification of other components of Sec biosynthesis and insertion machinery (e.g., SBP2, EFsec), SecS might become the most characteristic feature of the Sec trait in eukaryotes and archaea.

## Materials and Methods

### Materials.

[α-^32^P]ATP and [γ-^32^P]ATP (specific activity, approximately 6,000 Ci/mmol) and Hybond N^+^ nylon membranes were purchased from Amersham (http://www.amersham.com), ^3^H-serine (specific activity, 29.5 Ci/mmol) and ^14^C-pyruvate (specific activity, 19 mCi/mM) were from Perkin Elmer (http://www.perkinelmer.com), Ni-NTA agarose was from Qiagen (http://www.stratagene.com), and *pfu* polymerase and pBluescript II were from Stratagene (http://www.stratagene.com). pET32b vector (encoding the 109–amino acid thioredoxin with a His-tag) and BL21(DE3) competent cells were obtained from Novagen (EMD Biosciences, http://www.emdbiosciences.com), alkaline phosphatase from New England Biolabs (http://www.neb.com), T7 RiboMAX Express Large Scale RNA Production System and 3M filter paper from Whatman (http://www.whatman.com), and unlabeled amino acids, PEI TLC plates, and selenocystine from Sigma (http://www.signaaldrich.com). [(CH_3_)_3_SiO]_3_PSe was chemically synthesized [31]. All other reagents were commercial products of the highest grade available.

### Computational analyses.

A total of 50 completely or nearly completely sequenced eukaryotic genomes (Table S1) were analyzed for occurrence of selenoproteins by TBLASTN using the set of all known selenoproteins. Twenty-six organisms were found to contain selenoproteins and 24 organisms lacked these proteins. In organisms lacking selenoproteins, the Sec insertion machinery was also missing. To identify proteins with phylogenetic profiles corresponding to selenoproteins, annotated genes in D. melanogaster were used as a query dataset. BLAST homology analyses were used to scan genomic databases using the following criteria: E-value less than 1e^−06^ and length of the conserved region greater than 50 amino acids. Genes present in any of the organisms lacking selenoproteins were dismissed. The remaining genes were searched against selenoprotein-containing organisms to determine their occurrences. Top candidate genes were included in Table 1; they were present in 80% of selenoprotein-containing eukaryotes. These candidates were further manually analyzed for possible function.

A similar search strategy was carried out in archaea. A total of 27 archaeal genomes (Table S1), including three selenoprotein-containing organisms (M. jannaschii, *M. maripaludis,* and M. kandleri), were analyzed for gene occurrence using all annotated genes in M. jannaschii. Top candidate genes are included in Table 2; these proteins were present in all three selenoprotein-containing archaea and absent in other completed archaeal genomes. We used ClustalW to generate multiple sequence alignment. Phylogenetic trees were built with PHYLIP programs.

### Gene cloning and protein expression and purification.

The coding regions of *E. coli selA* and *selD*, mouse *secS*, *sps2*, *sps1,* and *C. elegans sps2* genes were amplified from BL21 genomic DNA or mouse liver cDNA or C. elegans total cDNA using *pfu* polymerase, respectively [20]. The resulting product was cloned into the pET32b vector at the NdeI-XhoI cloning sites in which the vector contained a His-tag immediately downstream of, and in frame with, the open reading frame. The Sec TGA codon in *sps2* was mutated to a Cys TGC codon using a site-directed mutagenesis kit (Stratagene), and the resulting gene product was designated mSPS2-Cys. The cDNA constructs were confirmed by sequencing and transformed into BL21(DE3) cells. Expression and purification of each protein were carried out as described [20]. For mSecS and SelA expression and purification, 1 nM PLP was added in the LB medium during expression and 5 μM PLP was added in the elution buffer during purification. The proteins were dialyzed against 1× TBS for 2 h and stored at −20 °C in 50% glycerol before use.

### Binding of tRNA^[Ser]Sec^ to mSecS.

Native tRNA^[Ser]Sec^ was purified and aminoacylated with serine and the seryl moiety phosphorylated as described [20]. Then 200 ng of purified mSecS containing a His-tag on its C-terminal was added in a total volume of 100 μl solution (20 mM Tris-HCl [pH 7.4], 0.01 mM EGTA, 1 mM DTT, 10 mM MgCl_2_, and 5 μg of yeast tRNA) and approximately 50 ng of purified tRNA^[Ser]Sec^ (with either serine or phosphoserine attached, or no amino acid) added, and the reaction was incubated for 30 min at room temperature. Anti-His agarose (10 μl) was added to pull down mSecS. After washing three times with 1 ml of 1× TBS/0.1% Tween, the agarose was suspended in 40 μl of TBE-urea loading buffer (90 mM Tris-HCl [pH 8.3], 64.6 mM boric acid, 2.5 mM EDTA, 3.5 M urea), and 5 μl of each sample was loaded onto a 15% TBE-urea gel. After electrophoresis and transfer of the RNA to a nylon membrane, RNA was detected by Northern blotting with the Sec tRNA probe [20]. Of each binding reaction, 2 μl had been removed immediately after the incubation period and electrophoresed along with the reaction samples for analysis by Northern blotting that served as a loading control.

### Dephosphorylation of *O*-phosphoseryl-tRNA^[Ser]Sec^ by SecS.

Native tRNA^[Ser]Sec^ was aminoacylated with serine and phosphorylated with [γ-^32^P]ATP using *O-*phosphoseryl-tRNA^[Ser]Sec^ kinase as described [20]. The ^32^P-labeled *O*-phosphoseryl-tRNA^[Ser]Sec^ was added to a 10-μl reaction mixture containing 50 mM Tris-HCl (pH 7.5), 20 mM DTT, 10 mM KCl, 10 mM MgCl_2_ and 1 μg of each purified protein. Reactions were carried out for 30 min at 37 °C. Following incubation, the tRNA was deacylated by adding an equal volume of 1 M Tris-HCl (pH 8.0) and incubating at 37 °C for 45 min. Reactions were then spotted onto 3M paper (Whatman), placed in a TLC chamber, and chromatographed for 8 h using a mixture of butanol/acetic acid/water (12:3:5). The chromatogram was then exposed to a PhosphorImager screen.

### In vitro ATP hydrolysis assay of selenophosphate synthetase and NMR spectroscopic analysis.

ATP hydrolysis assays were carried out in a volume of 10 μl with 40 mM HEPES (pH 7.4), 20 mM KCl, 10 mM MgCl_2_, 5 μCi of [α-^32^P]ATP, and 10 mM DTT and either with or without 0.25 mM selenide. After adding 0.3 mg/ml final concentration of each selenophosphate synthetase protein, reactions were incubated at 37 °C for 1 h under anaerobic conditions. Then 0.5 μl of each reaction was loaded onto PEI TLC plates, the plates were run in 0.8 M LiCl, and the developed TLC plates were exposed to a PhosphorImager screen. For NMR analysis, ATP hydrolysis reactions were carried out under anaerobic conditions in 3-mm NMR tubes in a total volume of 200 μl with 2 mM ATP instead of [α-^32^P]ATP. NMR tubes were sealed and incubated at 37 °C for 4 h prior to ^31^P NMR spectroscopic analysis [9].

### In vitro Sec biosynthesis.

Synthetic Sec tRNA was used in all biosynthetic reactions. Synthesis, purification, and aminoacylation of Sec tRNA were carried out as described [20]. All of the reactions were set up under anaerobic conditions before chromatographic analysis. For Sec biosynthesis, the selenium donor SeP was either generated from selenide by using mSPS2-Cys or hydrolyzed from chemically synthesized [(CH_3_)_3_SiO]_3_PSe [31]**.** For generating the selenium donor with mSPS2-Cys, a 10-μl reaction containing 50 mM NH_4_HCO_3_ (pH 7.6), 10 mM DTT, 2 mM MgCl_2_, 2 mM KCl, 2 mM ATP, and 2 μg of mSPS2-Cys with or without 1 mM selenide was preincubated at 37 °C for 1 h. The mSPS2-Cys reaction was added to 10 μl containing 50 mM Tris-HCl (pH 7.0), 20 mM DTT, 10 mM MgCl_2_, 2 μM PLP, 1.0 μg of purified mSecS, and approximately 5 μg (about 30,000 cpm) of either *O*-phospho-[^3^H]seryl-tRNA^[Ser]Sec^ or [^3^H]seryl-tRNA^[Ser]Sec^ and, in a positive control reaction, SelA in place of mSecS, and in a negative control reaction, thioredoxin (with a His-tag) in place of mSecS. Reactions were incubated at 37 °C for 2 h and then heated at 75 °C for 5 min, aminoacyl-tRNAs were deacylated [20], and 1 μl of an unlabeled amino acid mix (containing 12.5 mM concentration each of serine, *O-*phosphoserine, Sec, and alanine in 50 mM of KBH_4_) was added. Each reaction, along with several control lanes containing unlabeled amino acids and ^14^C-pyruvate, was chromatographed on Whatman 3M filter paper (45 × 60 cm) in ethanol/acetic acid/water (12:3:5) for 16 to 20 h. Then 1.0-cm strips were cut out of the dried chromatogram and counted in a liquid scintillation counter. The locations of each amino acid were determined by staining the lanes with unlabeled amino acids in 0.3% ninhydrin in acetone or by cutting out 1.0-cm strips of lanes with ^14^C-pyruvate and counting in a liquid scintillation counter. For direct use of SeP as a selenium donor, reactions contained the same components as above except 1 mM SeP was used in place of mSPS2-Cys reaction solutions and DTT was omitted from the mSecS reactions, since we found that the activity of mSecS is higher without DTT. SeP was generated by hydrolysis of 20 mM chemically synthesized [(CH_3_)_3_SiO]_3_PSe [31]. Reactions were incubated and analyzed as above.

For measurement of the SecS synthesis rate, reactions were carried out in a total volume of 10 μl of 50 mM Tris-HCl (pH 7.0), with 10 mM MgCl_2_, 10 mM KCl, 0.2 mM SeP, and 4 μg (approximately 120 pmol) of *O*-phospho-[^3^H]seryl-tRNA^[Ser]Sec^. Reactions were initiated by adding 2 μg (approximately 35 pmol) of mSecS and were stopped at specific time points between 0 and 80 min by boiling for 2 min and then deacylating and counting as described above.

## Supporting Information

Table S1 Eukaryotes and Archaea Used in the Computational Searches (41 KB PDF)

### Accession Numbers

GenBank (http://www.ncbi.nlm.nih.gov/Genbank) accession numbers for the sequences used in this paper are Homo sapiens (SecS_HS), Q9HD40; Mus musculus (SecS_MM), Q6P6M7; Drosophila melanogaster (SecS_DM), NP_649556; C. elegans (SecS_CE), Q18953; Methanococcus jannaschii (SecS_MJ), Q58027; Methanopyrus kandleri (SecS_MK), Q8TXK0. SelA sequences: *Escherichia coli* (SelA_EC), BAE77702; Geobacter sulfurreducens (SelA_GS), P61736. SelA-like sequences: Methanococcus jannaschii (SelA-like_MJ), Q57622; Methanopyrus kandleri (SelA-like_MK), AAM01835; *E. coli selA,* M64177; *selD,* M30184; mouse *secS,* AL049338; *sps2,* NM_009266; *sps1,* NM_175400; and *C. elegans sps2,* NM_070203. The numbers for other PLP-containing proteins are Methanococcus jannaschii Sep-tRNA:Cys-tRNA synthase (SepCysS_MJ), Q59072; Medicago truncatula Orn/Lys/Arg decarboxylase (COG1982_MT), ABE83138.1; Archaeoglobus fulgidus glutamate decarboxylase (COG0076_AF), O28275; and Thermococcus kodakarensis glycine/serine hydroxymethyltransferase (COG0112_TK), 05JF06.

## Abbreviations

DTT: dithiothreitol
mSecS: mouse selenocysteine synthase
mSPS1: mouse selenophosphate synthetase 1
mSPS2: mouse selenophosphate synthetase 2
mSPS2-Cys: mouse selenophosphate synthetase 2 containing an Sec (UGA)-to-Cys (UGC) mutation
PLP: pyridoxal phosphate
Sec: selenocysteine
SecS: selenocysteine synthase
SelA: *Escherichia coli* selenocysteine synthase
SelD: *E. coli* selenophosphate synthetase
SeP: monoselenophosphate
tRNA: transfer RNA
