# Supplementary material for: Biosynthesis of Selenocysteine on Its tRNA in Eukaryotes
Source: PLoS Biol. 2006 Dec 26;5(1):e4. doi: 10.1371/journal.pbio.0050004 (PMC1717018; doi:10.1371/journal.pbio.0050004)
Supplement: Supplementary file 1 [file pbio.0050004.st001.pdf]

Supplemental Information for *PLoS Biology*

Biosynthesis of Selenocysteine in Eukaryotes

Table S1. Eukaryotes and archaea used in the computational searches.[1]

Domain	Phyla	Organism	Selenoprotein-containing organism
**Eukaryota**			
	Viridiplantae	*Arabidopsis thaliana*	-
		Oryza sativa	-
	Chlorophyta	*Ostreococcus tauri*	+
		Chlamydomonas reinhardtii	+
	Apicomplexa	*Cryptosporidium hominis*	-
		Cryptosporidium parvum	-
		Plasmodium falciparum	+
		Plasmodium berghei	+
		Plasmodium yoelii	+
		Plasmodium chabaudi	+
	Dictyosteliida	*Dictyostelium discoideum*	+
	Diplomonadida	*Giardia lamblia*	-
	Entamoebidae	*Entamoeba histolytica*	-
		Entamoeba dispar	-
	Fungi	*Neurospora crassa*	-
		Aspergillus fumigatus	-
		Hypocrea jecorina	-
		Candida albicans	-
		Debaryomyces hansenii	-
		Eremothecium gossypii	-
		Kluyveromyces lactis	-
		Yarrowia lipolytica	-
		Saccharomyces cerevisiae	-
		Schizosaccharomyces pombe	-
		Cryptococcus neoformans	-
		Ustilago maydis	-
		Phanerochaete chrysosporium	-
		Encephalitozoon cuniculi	-
	Metazoa	*Ciona intestinalis*	+
		Danio rerio	+
		Takifugu rubripes	+
		Tetraodon nigroviridis	+
		Drosophila melanogaster	+
		Anopheles gambiae	+
		Bombyx mori	+
		Gallus gallus	+
		Xenopus tropicalis	+

		Caenorhabditis elegans	+
		Homo sapiens	+
		Mus musculus	+
		Rattus norvegicus	+
	Kinetoplastida	*Leishmania major*	+
		Trypanosoma brucei	+
		Trypanosoma cruzi	+
	Stramenopiles	*Thalassiosira pseudonana*	+
		Phytophthora ramorum	+
		Phytophthora sojae	+
	Alveolata	*Theileria parva*	-
		Paramecium tetraurelia	-
	Cryptophyta	*Guillardia theta*	-
**Archaea**			
	Desulfurococcales	*Aeropyrum pernix*	-
	Sulfolobales	*Sulfolobus acidocaldarius*	-
		Sulfolobus solfataricus	-
		Sulfolobus tokodaii	-
	Thermoproteales	*Pyrobaculum aerophilum*	-
	Archaeoglobales	*Archaeoglobus fulgidus*	-
	Halobacteriales	*Haloarcula marismortui*	-
		Halobacterium sp.	-
		Natronomonas pharaonis	-
	Methanobacteriales	*Methanosphaera stadtmanae*	-
		Methanothermobacter thermautotrophicus	-
	Methanococcales	*Methanocaldococcus jannaschii*	+
		Methanococcus maripaludis	+
	Methanomicrobiales	*Methanospirillum hungatei*	-
	Methanopyrales	*Methanopyrus kandleri*	+
	Methanosarcinales	*Methanococcoides burtonii*	-
		Methanosarcina acetivorans	-
		Methanosarcina barkeri	-
		Methanosarcina mazei	-
	Thermococcales	*Pyrococcus abyssi*	-
		Pyrococcus furiosus	-
		Pyrococcus horikoshii	-
		Thermococcus kodakaraensis	-
	Thermoplasmales	*Picrophilus torridus*	-
		Thermoplasma acidophilum	-
		Thermoplasma volcanium	-
	Nanoarchaeota	*Nanoarchaeum equitans*	-

[1]50 eukaryotes and 27 archaea were used in these searches.
